# Inherited burden for disease predisposition in diverse populations

**DOI:** 10.21203/rs.3.rs-6169692/v1

**Published:** 2025-03-10

**Authors:** Barış Kayaalp, Meltem Ece Kars, Yuval Itan, Ayşe Nazlı Başak, Jean-Laurent Casanova, Tayfun Özçelik

**Affiliations:** Bilkent University; Icahn School of Medicine at Mount Sinai; Icahn School of Medicine at Mount Sinai; Koç University; Rockefeller University; Bilkent University

**Keywords:** genetic disease, carrier frequency, genetic prevalence, public health genomics

## Abstract

**Background:**

Inherited burden for disease predisposition in diverse populations is an open question. American College of Medical Genetics and Genomics (ACMG) guidelines for variant classification, combined with large population variation databases, promise to provide valuable answers. We recently developed a robust ACMG-based automated variant classification tool and categorized the exome sequencing variants of 730,947 individuals from gnomAD.

**Methods:**

We leveraged the allele frequency information of variants in 3895 Genomics England PanelApp genes and identified 76,677 pathogenic (P) and 295,356 likely-pathogenic (LP) variants, expanding the ClinVar submissions nearly fivefold.

**Results:**

We found that, on average, an individual is born with 4.31 P or LP variants, of which 1.59 are compatible with a Mendelian condition, 1 in 12 presents with an actionable genotype, and a total of 372 genes are candidates for carrier screening. Furthermore, a genome-first approach revealed that the likelihood of having a genotype compatible with a disease is highest for congenital (1 in 2.24 individuals; 3.37 billion worldwide) followed by nervous (1 in 3.01; 2.39 billion), blood/immune (1 in 3.29; 2.04 billion), musculoskeletal/connective (1 in 3.65; 1.87 billion), skin (1 in 4.46; 1.62 billion), endocrine/metabolic (1 in 4.53; 1.62 billion), circulatory (1 in 7.26; 994 million), eye (1 in 7.62; 961 million), ear (1 in 8.39; 880 million), genitourinary (1 in 10.15; 750 million), neoplasm (1 in 16.01; 410 million), digestive (1 in 18.26; 312 million) and respiratory (1 in 47.72; 155 million) disorders.

**Conclusions:**

Evidence-based genetic epidemiology demonstrates the potential of personalized medicine for the implementation of early preventive measures and incentivization of lifestyle changes to enhance healthspan and lifespan. From a societal standpoint, this research demonstrates the importance of informing the public to decrease discrimination and social stigmatization associated with inherited diseases, as an overwhelming majority of individuals are expected to carry germ-line risk variants on average.

## Background

Whole-exome sequencing (WES) is a first-line diagnostic test and an integral component of research [1, 2]. Genomics England launched the PanelApp to facilitate accurate gene-to-phenotype associations [3]. To standardize the clinical interpretation of genetic variations, the American College of Medical Genetics and Genomics (ACMG) and the Association for Molecular Pathology have developed guidelines that rely on functional studies, *in silico* predictions, as well as population frequency and reported variations data [4]. This resulted in the designation of a variant as pathogenic (P), likely pathogenic (LP), variant of uncertain significance (VUS), likely benign (LB), or benign (B). To fully exploit this advancement, ACMG recommends clinicians implement surveillance of 81 secondary finding genes, as well as screening of all genes with a carrier frequency higher than 1 in 200 [5, 6]. However, variant classification from sequence data is an intricate process. To address this issue, we recently developed the Automated ACMG-based Variant Classifier (AAVC), which reaches a concordance rate of 99.3% with the Food and Drug Administration-approved expert variant curations [7].

These developments provide an unprecedented opportunity to compile a comprehensive account of the burden of inherited predisposition to disease using large-scale population data and variant prioritization methods. Indeed, several studies assessed the extent of previously reported pathogenic and high-impact variants in disease genes [8–19]. However, they are short of evaluating the entire reported allelic diversity present in large datasets in diverse populations. Also, they often disregard the disease mechanism, functional studies, and ACMG classification. Therefore, we classified all variants in the Genome Aggregation Database (gnomAD), representing 730,947 exomes across eight genetic ancestry groups, using AAVC, and calculated the per-individual burden of disease-associated variants, carrier frequency (CrF), and genetic prevalence (GP) for each known disease gene [20]. In addition, we resorted to the International Classification of Diseases, Tenth Revision (ICD-10), and calculated genetic prevalence according to disease groups.

## Methods

### Selection of disease gene list

The Genomics England PanelApp gene list was downloaded from the University of California Santa Cruz Genome Browser on May 23, 2024 [3, 21]. PanelApp prioritizes genes with a Mendelian pattern of causation appropriate for reporting in a diagnostic setting, with the exclusion of intermediate and low penetrance genes. The “green” genes, indicating high-level evidence of disease-genes association, were prioritized, yielding 3970 disease genes across 303 panels. The breakdown of the inheritance for the 3970 genes was 2013 autosomal recessive, 1425 autosomal dominant, 256 both autosomal dominant and recessive, 148 X-linked recessive, 65 X-linked dominant, 35 mitochondrial, one pseudoautosomal dominant, one pseudoautosomal recessive, and one Y-linked. For 25 genes, the inheritance pattern was not ascertained. The sole disease mechanism for 13 of the genes was repeat expansion, and for 2 genes, the disease mechanism could not be ascertained. Short variants of the genes leading to disease by only repeat expansion were also removed since such variations were absent from the gnomAD exome data. We subjected 3895/3970 genes for downstream analyses, excluding 75 (60 had either undefined or mitochondrial inheritance patterns, 2 without a clearly defined disease mechanism, and 13 of them leading to a phenotype with repeat expansion) (Additional File 1: Table S1).

### Determination of disease mechanism

The disease mechanism of a gene can be loss of function (LoF), gain of function (GoF), or LoF + GoF. LoF + GoF genes could lead to a similar phenotype in both LoF and GoF direction, such as *STAT1*; or a new one, such as *ANO5*, which leads to LoF (Miyoshi muscular dystrophy) or GoF (Gnathodiaphyseal dysplasia) (38, 39). To curate a list of GoF and LoF + GoF disease genes, we parsed OMIM (https://omim.org/) in September 2024 for “gof,” “gain-of-function,” and “gain of function” prompts. This yielded 600 genes, of which 488 were in the PanelApp gene list. Preliminary GoF gene status was given to 488 genes, and each gene was manually reviewed and curated to determine the disease mechanism. This resulted in 146 GoF, 161 LoF + GoF genes. Twenty nine out of 161 LoF + GoF genes had dominant GoF and recessive LoF phenotype; for example, GoF mutations in *ORAI1* lead to dominant tubular aggregate myopathy, while LoF mutations lead to a recessive immunodeficiency [24]. Loss of function-associated scores provided by AAVC, PVS1, were removed for GoF phenotypes. Although end-truncating variants can lead to a GoF effect, *NOTCH2* related skeletal disorders, there is no established guideline for the detection of such cases [25]. In summary, 146 genes were GoF, 161 genes GoF + LoF, and 3588 genes LoF, totaling 3895 genes (Additional File 1: Table S1).

### Distribution of genes to disease groups

We nest distributed the panels into 13 disease categories based on the ICD-10 grouping system, allowing for the inclusion of a single gene in multiple disease groups. Three panels, “Severe Paediatric Disorders, 2563 genes” “Paediatric disorders – additional genes, 49 genes” and “Additional findings health related – children, 7 genes”, combine multiple panels and therefore did not bin into a disease group. The total number of the genes in these three panels is 2589 but not 2619, since 30 genes are shared. While 2562/2589 of these genes were also present in other panels, 27 were unique. Therefore, they were matched to disease groups based on the associated phenotypes. The number of ICD-10 disease groups assigned for a gene ranged from nine for a single gene, *KMT2D*, to one for 1283 genes. For example, *CFTR* was assigned to five different disease groups: blood/immune, congenital, digestive, endocrine/metabolic, and respiratory (Additional File 1: Table S1).

### Variant selection, annotation, and classification

gnomAD exome v4.1.0 variant call format (VCF) files containing 730,947 exomes were downloaded on May 23, 2024 (https://gnomad.broadinstitute.org/data#v4-variants). The data contains Allele Count (AC), and Allele Number (AN) information for eight genetic ancestries, African/African American (afr), Admixed American (amr), Ashkenazi Jewish (asj), East Asian (eas), Finnish (fin), Middle Eastern (mid), non-Finnish European (nfe) and South Asian (sas). We selected high-quality variants that passed the variant quality score recalibration (VQSR) filter (PASS) and covered more than 50% of individuals for corresponding ancestries for 3895 disease genes, leaving 13,535,162 variants. Ensembl Variant Effect Predictor release 111 was used for the pre-annotation required for AAVC [26]. AAVC classifies variants into seven broad categories of P (odds of pathogenicity > 99%), LP (90%), VUS-H (67.5%), VUS-M (41%), VUS-L (14%), LB (10%), and B (< 1%) (Additional File 2: Supplementary Notes) [27].

### Statistical analysis of carrier frequency and genetic prevalence

Allele frequency (AF) for a variant was calculated as AF=AC/AN. For autosomal and pseudo-autosomal genes, Cumulative allele frequency (CAF) for a gene was calculated by adding allele frequencies of each P, P+LP, or P + LP+VUS-H variant, assuming they were independently assorted. Carrier frequency (CrF), which denotes the proportion of individuals in a population who have a single copy of a genetic variant, was calculated utilizing CAF and Hardy-Weinberg Equilibrium:

CrF=2*CAF*(1-CAF)

The sum of homozygote and compound heterozygote frequency (HomF) was calculated by squaring CAF. The genetic prevalence of each compound heterozygote (CompHet) was calculated by using the allele frequency of each allele with the formulation below:

$$\mathrm{CompHet}\;={\left(AF_{i}+AF_{ii}\right)}^{2}-AF_{i}^{2}-AF_{ii}^{2}=2\;*\;AF_{i}\;*\;AF_{ii}$$

where ${AF}_{i}$ and ${AF}_{ij}$ are the allele frequencies of 2 different alleles.

Genetic prevalence (GP), which denotes the proportion of a population that has a causal genotype for a genetic disorder, was assumed to be CrF for AD or AD/AR genes and HomF for AR genes. For X-linked recessive genes, we calculated GP as (CAF*(CAF+1))/2 and for X-linked dominant genes as (1-CAF)*CAF+(CAF*(CAF+1))/2. The likelihood of an individual not carrying any variant was calculated with CrF of all genes:

$$\mathrm{Non}-\mathrm{Carrier}\;\mathrm{Likelihood}=\prod_{i=1}^{n}\;\left(1-CrF_{i}\right)$$

where n is the number of all 3895 disease genes.

For cumulative GP of ICD-10 disease groups, each gene was counted once for each inheritance category they have; for example, *ANO5*, both dominant, gnathodiaphyseal dysplasia (GoF), and recessive, muscular dystrophy (LoF) was in Musculoskeletal/Connective group. Thus, the GP for both phenotypes was summed, while the cumulative GP in the Musculoskeletal/Connective group was calculated. A similar approach was adopted to calculate the cumulative GP for all genes, and each gene was counted once for both dominant and recessive phenotypes, while for cumulative CrF, each gene was counted once regardless of inheritance.

To test the correlation between reported CrF (rCrF) and estimated CrF for autosomal recessive diseases, we used a linear regression model for log-transformed values. rCrF is calculated based on the reported prevalence (rP) of a given Mendelian disease using the following equation:

$$rCrF=2\;*\;\sqrt{rP}\;*\;(1-\sqrt{rP})$$

For the list of all curated rCrF from the literature, please see Additional File 1: Table S2. For all genetic ancestry groups, population size data for 2023 is presented in Additional File 1: Table S3.

### Gene Constraint Metrics

pLI, LOEUF, and missense and synonymous Z scores were downloaded from https://gnomad.broadinstitute.org/data#v4-constraint for gnomAD version 4. S_het_ scores were downloaded for Regeneron Genetics Center Million Exome cohort from a previous publication (13). Genes were deemed constrained for different metrics with different thresholds, pLI > 0.9, LOEUF < 0.6, S_het_ >0.073, Missense Z > 3.09, Synonymous Z > 3.71 [13, 28, 29]. Although the original recommendation for LOEUF was < 0.35, it was updated to < 0.6 for gnomAD version 4 (https://gnomad.broadinstitute.org/news/2024-03-gnomad-v4-0-gene-constraint/#loeuf-guidance) (Additional File 2: Supplementary Notes).

## Results

### Variant Classification

**Figure 1:**
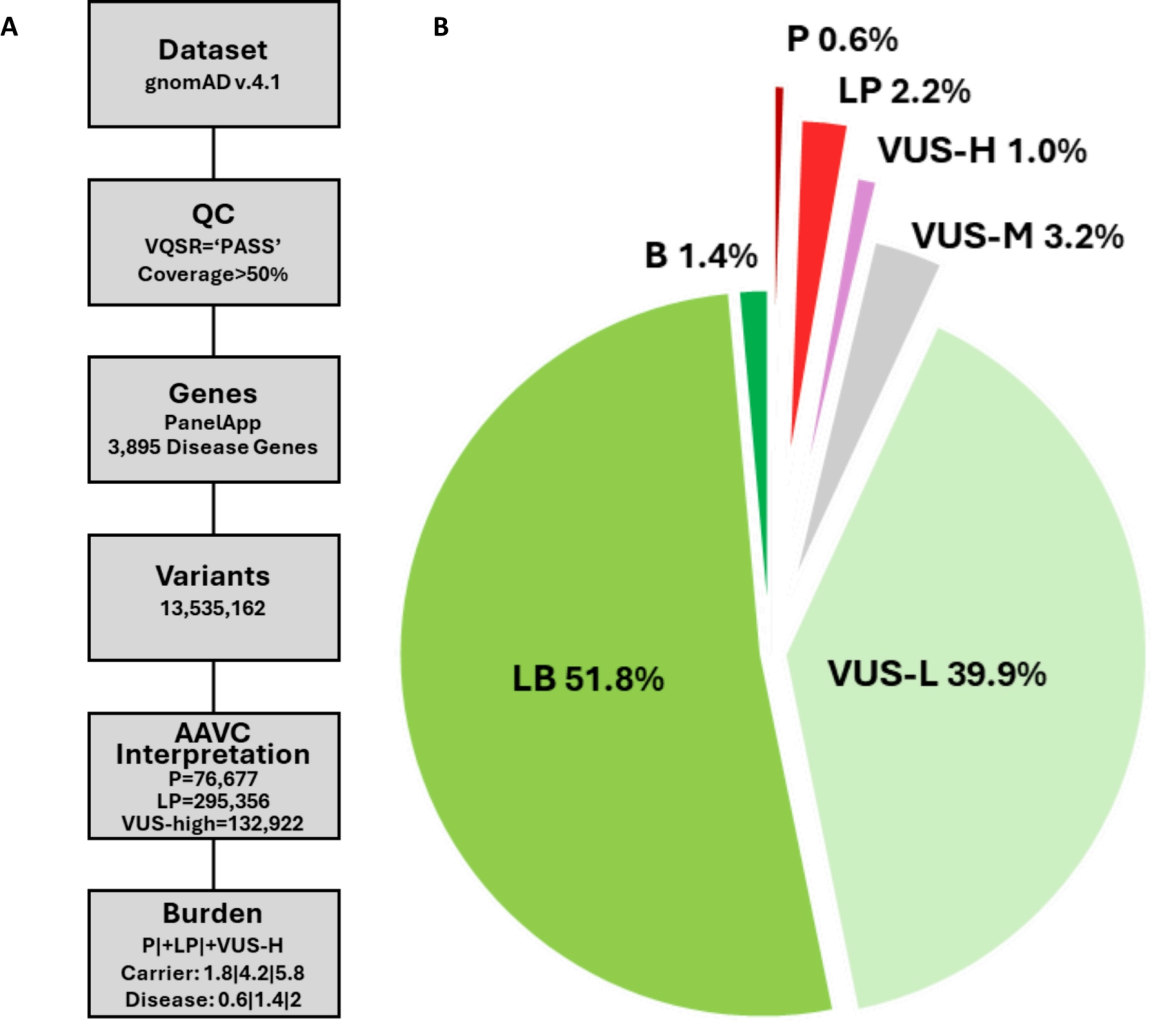
Variant classification and distribution. **A** High-quality variants were selected from gnomAD that passed the VQSR filter and covered more than half of the individuals. Variants of genes with a high level of evidence for disease association, and therefore labelled “green” in PanelApp, were selected. We used AAVC to classify variants according to ACMG guidelines. We next calculated the burden of each genetic disease based on the allele frequency information of P+LP+VUS-H variants in gnomAD. **B**Distribution of classified variants. P+LP variants, corresponding to 2.8%, were prioritized for downstream burden analyses.

The PanelApp data lists 3970 disease genes. However, 60 of them had either undefined or mitochondrial inheritance patterns, 2 of them without a clearly defined disease mechanism, and 13 of them leading to a phenotype with repeat expansion. Therefore, we excluded these 75 genes and selected 3895 with well-defined autosomal dominant (AD), autosomal recessive (AR), and X-linked (XL) phenotypes for downstream analyses (Additional File 1: Table S1). Two genes from the pseudoautosomal regions of the X and Y chromosomes, *SHOX* and *CSF2RA*, were in the AD and AR groups, respectively. Next, we subjected the variants of the 3895 genes from the gnomAD v.4.1.0 data to quality control steps followed by AAVC classification. This yielded 76,677 P (0.6%), 295,356 LP (2.2%), 132,922 VUS-high (VUS-H) (1.0%), 431,541 VUS-mid (VUS-M) (3.2%), 5,396,423 VUS-low (VUS-L) (39.9%), 7,012,152 LB (51.8%) and 190,091 B (1.4%) variants (Fig. 1A, Fig. 1B and Additional File 2: Fig. S1). ClinVar submissions corresponded to 793,311 variants (5.9%). Functional studies reported in the literature covered 19,933 (0.15%) variants (Additional File 2: Supplementary Notes and Additional File 1: Table S4).

### Carrier frequency and genetic prevalence

**Figure 2:**
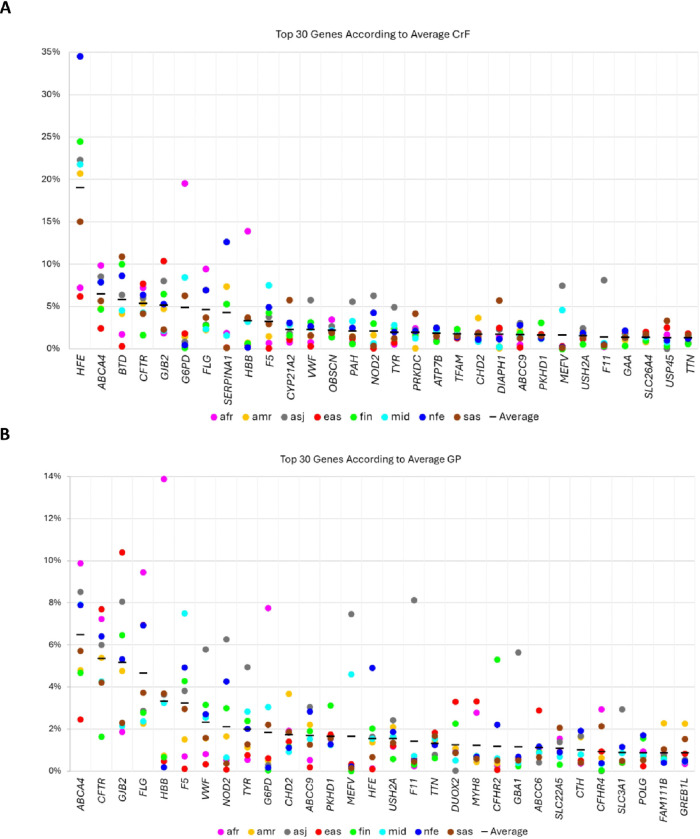
Top 30 genes according to CrF and GP. P+LP variants were prioritized across eight gnomAD genetic ancestry groups. The x-axes show the gene symbols and the y-axes show the **A** CrF and **B** GP. The ancestries are afr (African), amr (Admixed American), asj (Ashkenazi Jewish), eas (East Asian), fin (Finnish), mid (Middle Easterner), nfe (non-Finnish European) and sas (South Asian).

We calculated CrF and GP for each gene for the three sets of variants, P, P + LP, and P + LP + VUS-H, across eight genetic ancestries (Additional File 2: Supplementary Notes, and Additional File 1: Tables S5 and S6). The top genes with the highest CrF for P + LP variants were *G6PD* (XL, favism) for Africans (1/5 individuals; 255 million individuals); *GJB2* (AD, palmoplantar keratoderma) for East Asians (1/10; 198 million); and *HFE* (AR, hemochromatosis) for non-Finnish Europeans (1/3; 335 million), Finnish (1/4; 1.37 million), Ashkenazi Jews (1/4; 3.51 million), Middle Easterners (1/5; 111 million), Admixed Americans (1/5; 151 million) and South Asians (1/7; 293 million), which was driven by two missense variants with penetrance as low as 4.5% [30] (Fig. 2A and Additional File 1: Tables S3 and S6). We investigated the correlation between reported CrF and calculated CrF for a given autosomal recessive disease, and they were better correlated for Europeans (R = 0.67, p < 0.001) compared to non-Europeans (R = 0.44, p = 0.028), probably due to the underrepresentation of non-Europeans in epidemiological studies (Additional File 2: Supplementary Notes and Fig. S2 and Additional File 1: Table S2). To further investigate this discrepancy, we conducted a case study on one of the best-documented Mendelian genes, *CFTR* with a 1 in 25 carriership for Europeans, using the *CFTR*-France database [31, 32]. We calculated that 1 in 21 individuals carries a hypomorphic and 1 in 27 cystic fibrosis-causing variants, translating to an overall carriership rate of 1 in 12, suggesting hypomorphic alleles as a contributor to the discrepancy (Additional File 2: Supplementary Notes and Additional File 1: Table S7).

When the genes were ordered based on GP, the most frequently observed were *HBB* (AD, hemoglobinopathies) for Africans (1/7; 182 million); *CFTR* (AD/AR, cystic fibrosis and related disorders) for Admixed Americans (1/19; 39 million); *ABCA4* (AD/AR, retinal disorders) for Ashkenazi Jews (1/12; 1.34 million), Middle Easterners (1/13; 40 million), non-Finnish Europeans (1/13; 77 million) and South Asians (1/18; 111 million); and *GJB2* (AD, palmoplantar keratoderma) for East Asians (1/10; 198 million) and Finnish (1/15; 0.36 million) (Fig. 2B). GP and CrF data for all disease genes can be explored using the interactive table in Additional File 3: Interactive table for all genetic ancestry groups.

**Figure 3:**
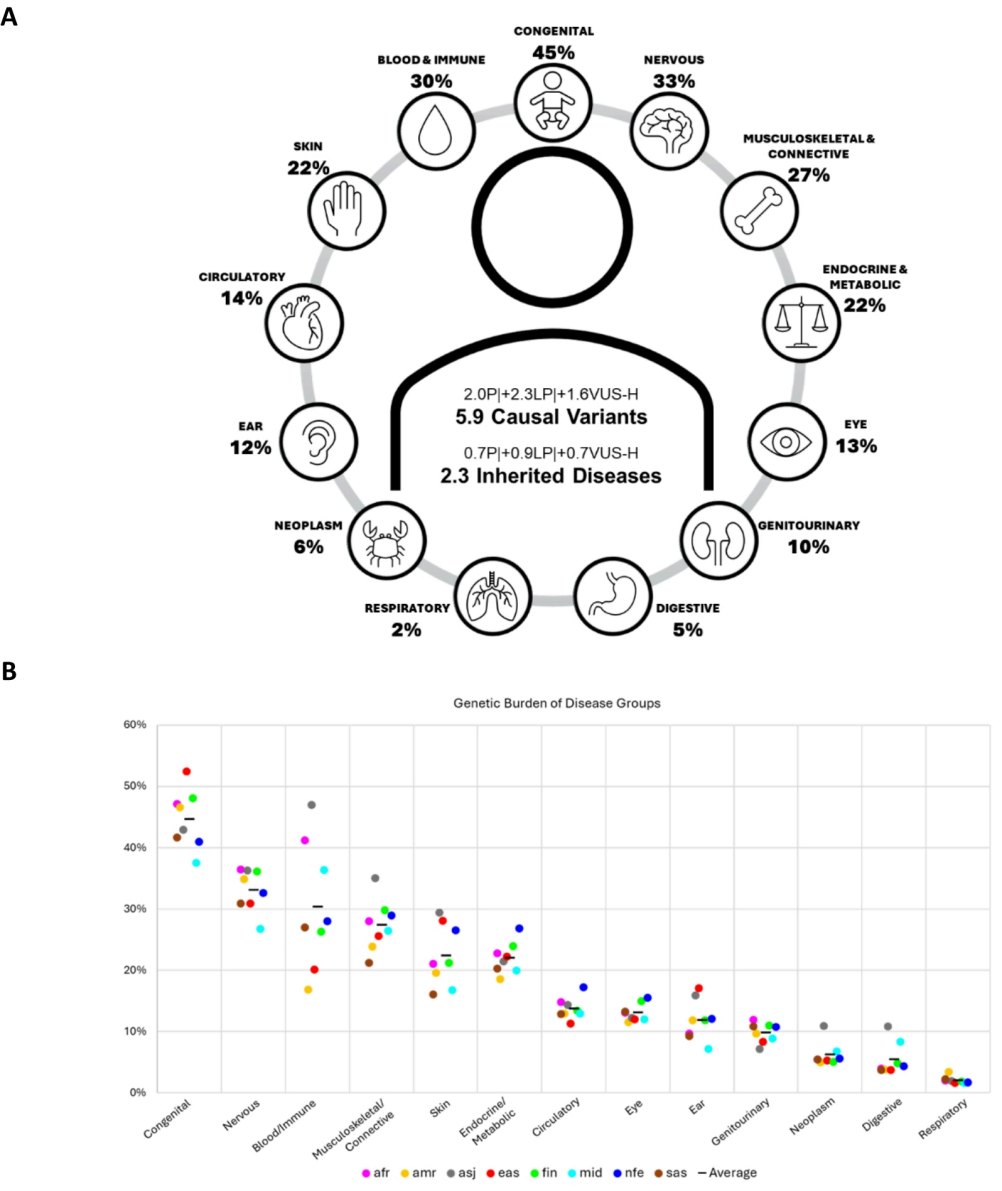
Genomic burden of inherited predisposition to diseases. **A** P+LP+VUS-H variants per individual for 3895 disease genes, and GP of P+LP variants according to ICD-10-based disease groups on average for the eight genetic ancestries. **B** GP distribution for P+LP variants according to ICD-10-based disease groups for all genetic ancestries. The x-axis shows the 13 different disease groups, and the y-axis shows the GP. The ancestries are afr (African), amr (Admixed American), asj (Ashkenazi Jewish), eas (East Asian), fin (Finnish), mid (Middle Easterner), nfe (non-Finnish European) and sas (South Asian).

Next, we calculated the probability of an individual carrying any P, LP, or VUS-H variant based on CrF. The mean across all genetic ancestries was 2.01 for P, 4.31 for P + LP, 5.88 for P + LP + VUS-H. Regarding the cumulative GP of the genes, an individual had, on average, 0.71 P, 1.59 P + LP, and 2.32 P + LP + VUS-H genotype compatible with a heterozygous dominant, homozygous recessive, or hemizygous X-linked disorder. The probability for an individual not to carry any P + LP variant was 1 in 79 (1.27%; 99 million) and P + LP + VUS-H variant was 1 in 387 (2.58‰; 18 million) (Fig. 3A, Additional File 1: Tables S3 and S8).

### Inherited burden according to disease groups

We adopted a genome-first approach to investigate the inherited burden for 13 disease groups compiled from ICD-10 codes and employed a bipartite matching approach between genes and phenotypes. Of the 3895 disease genes, 1283 (33%) were in a single disease group, 1378 (35%) in two disease groups, and the remaining 1234 (32%) in more than two disease groups, and the highest, *KMT2D* (Kabuki syndrome), in 9 disease groups. Next, we assessed the burden of each disease group by summing the GP of genes using P + LP variants across eight genetic ancestries. The average ranged from 1 in 2 to 1 in 48: congenital (45%; 3.37 billion), nervous (33%; 2.39 billion), blood/immune (30%; 2.04 billion), musculoskeletal/connective (27%; 1.87 billion), skin (22%; 1.62 billion), endocrine/metabolic (22%; 1.62 billion), circulatory (14%; 994 million), eye (13%; 961 million), ear (12%; 880 million), genitourinary (10%; 750 million), neoplasm (6%; 410 million), digestive (5%; 312 million) and respiratory (2%; 155 million) (Fig. 3A and B, Additional File 1: Tables S3 and S8).

The top 3 genes according to CrF in 13 disease groups and their associated disorders are listed in Table 1. When we assessed the genetic burden in each genetic ancestry separately, the likelihood of having a genetic disease was highest for congenital in East Asians (53%; 1 billion), Finnish (48%; 2.69 million), Africans (47%; 617 million), Admixed Americans (47%; 340 million), South Asians (42%; 815 million), non-Finnish Europeans (41%; 398 million), Middle Easterners (38%; 191 million) and for blood/immune in Ashkenazi Jews (47%; 7 million). The lowest burden for all genetic ancestries was for the respiratory disease group. When we adjusted the burden for dominant disorders in each disease group for all genetic ancestries, skin, blood/immune, and ear diseases ranked the highest, and nervous, neoplasm and congenital diseases ranked the lowest. A lower prevalence of P or LP variants per dominant gene suggested haploinsufficiency, which was also observed as a negative selection against the high-impact variants for nervous, neoplasm and congenital disease genes, as supported by gene constraint metrics. Moreover, genes that were classified under more than one disease group were under a stronger selection for high-impact and missense variants compared to those in only a single disease group (Additional File 2: Supplementary Notes and Fig. S3; Additional File 1: Tables S9 and S10).

### Secondary finding genes

ACMG listed 81 genes as actionable and recommends reporting all P + LP variants for 80 of them plus only the C282Y variant for *HFE* [5]. When SNPs and short indels of the 81 ACMG secondary finding genes were analyzed, cumulative CrF for P + LP variants across genetic ancestries ranged from 15.1% for East Asians to 34.9% for non-Finnish Europeans. The highest CrF after averaging across eight genetic ancestries was for *BTD* (AR; biotinidase deficiency; 1/17; 382 million), followed by *HFE (*AR; hemochromatosis; 1/28; 471 million) and *ATP7B* (AR; Wilson disease; 1/54; 137 million) (Additional File 1: Tables S3, S8 and S11).

Next, we investigated the cumulative GP, which ranged from 6.9% (Finish) to 11.8% (African) for the 81 genes. The highest GP was observed for *TTN* (AD; dilated cardiomyopathy; 1/76; 117 million), *TTR* (AD; familial transthyretin amyloidosis; 1/89; 51 million), and *LDLR* (AD; familial hypercholesterolemia; 1/196; 46 million) across eight genetic ancestries. With respect to disease groups as defined by the ACMG Secondary Findings Working Group, the burden was highest for cardiovascular (1/21; 404 million) followed by cancer (1/57; 121 million), miscellaneous (1/69; 122 million) and inborn errors of metabolism (1/217; 45 million) phenotypes (Additional File 1: Tables S3, S8 and S11) [5, 6].

### Candidate genes for screening

ACMG recommends screening for genetic disorders with a carrier frequency higher than 1 in 200 for all couples [6]. The current list contains 286 genes selected based on reported P + LP variants [6, 17, 33]. When we used ACMG guidelines for variant classification, 86 additional genes became candidates for screening, thus increasing the number to 372. Different sets of genes met the ACMG screening criteria across the eight genetic ancestries, and 13 genes were shared by all of them (Additional File 2: Fig. S4 and Additional File 1: S12).

## Discussion

Determining the true carrier frequency and genetic prevalence of an inherited disease, especially that of a rare disorder, is a significant challenge that requires nationwide systemic records for epidemiological statistics. Moreover, misdiagnosis represents an obstacle to frequency calculations, primarily for diseases such as Wilson disease, with a correct diagnostic rate as low as 7% [34]. Therefore, the gene-centric approach presented here provides a unique perspective on the inherited burden for P + LP variants in 3895 known disease-causing genes across eight genetic ancestries. After standardizing the variant classification process by using AAVC, we observed that an individual carries, on average, 4.31 P + LP variants, of which 1.59 are compatible with a heterozygous dominant, homozygous recessive, or hemizygous X-linked disorder, 1 in 12 individuals carry an actionable genotype, and 372 genes are candidates for carrier screening.

The effects of heterozygote advantage and population bottleneck were observed as CrF differences across different genetic ancestries. For example, high CrF of *HBB* and *G6PD* in Africans can be attributed to malaria resistance. Similarly, high frequency of *MEFV* variants in Middle Easterners and Ashkenazi Jews have been proposed to protect against *Yersinia pestis* infections, *CFTR* and *GJB2* variants against diarrhea, and *CYP21A2* variants have been hypothesized to decrease mortality from pneumonia [35–38]. *DHCR7* variant carriers have shown to exhibit higher levels of vitamin D, which might explain its high prevalence in non-Finnish Europeans [39]. On the other hand, *ASPA*, *ELP1*, *G6PC1*, and *HEXA* in the Ashkenazi Jews and *AGA*, *BCS1L*, *CLN5*, and *SLC17A5* in the Finnish, had higher CrFs probably due to population bottlenecks [40, 41]. We noted discrepancies between the reported and calculated CrFs, which probably arises from inbreeding, isolation, assortative mating, incomplete penetrance, locus heterogeneity, linkage disequilibrium, environmental factors for complex diseases, and under- or over-estimated frequencies due to false negatives or false positives [11, 42]. This was further exacerbated by the underrepresentation of non-Europeans in gnomAD, and a low number of epidemiological studies for such populations, which led to less accurate estimations for CrF and a higher discordance between the reported and calculated CrF. Incomplete penetrance likely contributed to the discrepancy between GP and reported prevalence in certain AD diseases such as *TGIF1-*associated holoprosencephaly, or hypomorphic variants in AR phenotypes like cystic fibrosis or hemochromatosis, particularly when the least conservative variant set (P + LP + VUS-H) was used [43].

When the burden was assessed according to ICD-10-based groups, the highest was for congenital diseases, followed by nervous, blood/immune, musculoskeletal, skin, and endocrine/metabolic diseases when all genetic ancestries were considered. By incorporating variant detection strategies that extend beyond short variant discovery to include large insertions and deletions, copy number variations, and pathogenic repeat expansion disorders, which are the prominent causes of diseases such as Duchenne muscular dystrophy, alpha thalassemia, spinal muscular atrophy, and Huntington’s disease, the estimations of inherited burden will become more accurate. The Impact of Genomic Variation on Function Consortium is expected to further advance non-coding, regulatory, and missense/ in-frame indel variant classifications [44].

## Conclusions

We anticipate that our findings will serve as a valuable resource for epidemiological studies as well as decision-making processes regarding the screening, diagnosis, and treatment of inherited diseases. From an individual’s perspective, one can learn about their genetic risk and, in some cases, have an opportunity to take preventive measures or initiate lifestyle modifications. We found that 1 in 12 individuals, corresponding to 635 million, carry a P + LP variant in the 81 ACMG-recommended actionable genes, which may have profound implications for healthspan and lifespan. For example, a recent study demonstrated that the presence of an actionable genotype in a cancer-related gene was associated with a three-year reduction in survival compared to noncarriers, and most deaths among carriers were attributed to cancer-related conditions [19]. From a health policy perspective, frequency estimates of certain genetic diseases can facilitate health economics analysis. Our findings can be utilized to identify candidate genes for population-specific genetic screening tests, which would benefit the equitable carrier screening of parents or newborns. As the cost of sequencing decreases, it can be foreseen that sequencing every newborn at birth will soon be feasible. A gene-centric approach that incorporates reverse phenotyping in a clinical setting promises to improve diagnostic accuracy and, thus, precision medicine interventions significantly [45]. Lastly, from a societal standpoint, we hope this research will decrease discrimination and social stigmatization associated with inherited diseases, as an overwhelming majority of individuals are expected to carry germ-line risk variants on average.

## Figures and Tables

**Table 1:** The top three genes of ICD-10-based disease groups.

Disease Group	Gene	I*	OMIM	CrF (1/x)^†^	GP (1/x)^†^
Congenital	*DHCR7*	AR	Smith-Lemli-Opitz syndrome (270400)	101|87|75	22K^‡^|19K|16K
	*ABCC6*	AR	Pseudoxanthoma elasticum (264800)	120|89|53	33K|22K|9K
	*CEP290*	AR	Bardet-Biedl syndrome 14 (615991)	165|132|121	95K|63K|52K
Nervous	*SPG7*	AD	Spastic paraplegia 7 (607259)	142|126|116	142|126|116
	*CHD2*	AD	Developmental/epileptic encephalopathy (615369)	105K|15K|7K	105K|15K|7K
	*NEB*	AR	Arthrogryposis multiplex congenita 6 (619334)	234|133|118	183K|62K|48K
Blood/Immune	*G6PD*	XLR	Favism (300908)	30|20|20	78|55|53
	*F5*	AD	Thrombophilia 2 (188055)	32|31|30	32|31|30
	*HBB*	AD	Thalassemia, beta (613985)	37|30|30	37|30|30
Musculoskeletal/Connective	*ALPL*	AD	Hypophosphatasia (146300)	155|131|120	27K|24K|22K
	*ANO5*	AR	Muscular dystrophy, 12 (611307)	183|105|96	81K|36K|31K
	*OBSCN*	AR	Rhabdomyolysis, susceptibility to, 1 (620235)	196|45|42	128K|7K|6K
Skin	*FLG*	AD	Dermatitis, atopic 2 (605803)	37|21|21	37|21|21
	*TYR*	AR	Albinism, oculocutaneous, type IB (606952)	56|50|44	8K|7K|6K
	*OCA2*	AR	Albinism, brown oculocutaneous (203200)	141|79|54	48K|17K|9K
Endocrine/Metabolic	*HFE*	AR	Hemochromatosis, type 1 (235200)	5|5|5	64|64|64
	*BTD*	AR	Biotinidase deficiency (253260)	18|17|17	805|771|764
	*PAH*	AR	Phenylketonuria (261600)	49|47|32	6K|6K|2.5K
Circulatory	*TTN*	AD	Cardiomyopathy, 1G (604145)	283|76|58	283|76|58
	*FKTN*	AR	Cardiomyopathy, dilated, 1X (611615)	414|365|359	148K|144K|143K
	*KCNQ1*	AD	Long QT syndrome 1 (192500)	692|409|223	692|409|223
Eye	*ABCA4*	AD/AR	Retinitis pigmentosa 19 (601718)	20|15|13	1.3K|775|560
	*CNGB3*	AR	Achromatopsia 3 (262300)	167|124|67	56K|42K|14K
	*EYS*	AR	Retinitis Pigmentosa 25 (602772)	180|100|73	93K|34K|15K
Ear	*GJB2*	AD	Deafness, 3A (601544)	20|19|19	20|19|19
	*SLC26A4*	AR	Deafness, 4 (600791)	97|72|67	32K|19K|16K
	*OTOG*	AR	Deafness, 18B (614945)	230|111|108	155K|44K|42K
Genitourinary	*SLC12A3*	AR	Gitelman syndrome (263800)	132|96|88	42K|24K|21K
	*PKHD1*	AR	Polycystic kidney disease 4 (263200)	162|60|41	162|60|41
	*NPHS1*	AR	Nephrotic syndrome, type 1 (256300)	233|186|157	63K|56K|49K
Neoplasm^§^	*BRCA2*	AD	Breast cancer (114480)	239|217|209	239|217|209
	*ATM*	AD	Breast cancer (114480)	350|245|228	350|245|228
	*BRCA1*	AD	Breast-ovarian cancer, familial, 1 (604370)	363|343|335	363|343|335
Digestive	*ATP7B*	AR	Wilson disease (277900)	83|54|43	22K|10K|7K
	*ALDOB*	AR	Fructose intolerance (229600)	166|150|140	79K|65K|62K
	*ABCC2*	AR	Dubin-Johnson syndrome	177|129|119	75K|49K|43K
Respiratory	*CFTR*	AR	Cystic fibrosis (219700)	26|19|9	2.2K|1.2K|286
	*SERPINA1*	AR	Emphysema due to AAT deficiency (613490)	59|23|23	7K|1.K|1K
	*SFTPB*	AR	Surfactant metabolism dysfunction, 1 (265120)	3.2K|1.2K|1.1K	23M^‡^|4M|4M

* : Inheritance

† : CrF and GP in the order of P|P + LP|P + LP + VUS-H variants

‡ : K, thousand, M, million

§ : The neoplasm disease group selects genes based on Genetic Prevalence instead of Carrier Frequency.

## Data Availability

gnomAD v4.1.0 allele frequency data is available at https://gnomad.broadinstitute.org/data. AAVC is freely available at https://aavc.bilkent.edu.tr/ All data generated during this study are included in the supplementary data and files; raw data for variant classifications is available at https://zenodo.org/records/14842663 and GP and CrF data for all disease genes can be explored using the interactive table in Additional File 3: Interactive Table for all genetic ancestry groups.

## Abbreviations

AAVC: Automated American College of Medical Genetic and Genomics-based Variant Classifier
AC: Allele count
ACMG: American College of Medical Genetic and Genomics
AD: autosomal/pseudoautosomal dominant
AF: Allele frequency
afr: African
amr: Admixed American
AN: Allele number
AR: autosomal/pseudoautosomal recessive
asj: Ashkenazi Jewish
B: Benign
CAF: Cumulative allele frequency
CompHet: Genetic prevalence of compound heterozygotes
CrF: Carrier Frequency
eas: East Asian
fin: Finnish
gnomAD: Genome Aggregation Database
GoF: Gain of Function
GP: Genetic Prevalence
HomF: Homozygote and compound heterozygote frequency
ICD-10: International Classification of Diseases, Tenth Revision
LB: Likely benign
LoF: Loss of Function
LP: Likely pathogenic
mid: Middle Easterner
nfe: non-Finnish European
P: Pathogenic
rCrF: Reported Carrier frequency
rP: Reported disease prevalence
sas: South Asian
VCF: Variant call format
VQSR: Variant quality score recalibration
VUS: Variant of uncertain significance
VUS-H: Variant of uncertain significance-high
VUS-L: Variant of uncertain significance-low
VUS-M: Variant of uncertain significance-mid
WES: Whole Exome Sequencing
XL: X-linked
